# Development of Functional Human NK Cells in an Immunodeficient Mouse Model with the Ability to Provide Protection against Tumor Challenge

**DOI:** 10.1371/journal.pone.0008379

**Published:** 2009-12-21

**Authors:** Amanda Kwant-Mitchell, Elishka A. Pek, Kenneth L. Rosenthal, Ali A. Ashkar

**Affiliations:** Centre for Gene Therapeutics, Department of Pathology and Molecular Medicine, McMaster University Health Sciences Centre, Hamilton, Ontario, Canada; Karolinska Institutet, Sweden

## Abstract

Studies of human NK cells and their role in tumor suppression have largely been restricted to *in vitro* experiments which lack the complexity of whole organisms, or mouse models which differ significantly from humans. In this study we showed that, in contrast to C57BL/6 Rag2^−/−^/γ_c_^−/−^ and NOD/Scid mice, newborn BALB/c Rag2^−/−^/γ_c_^−/−^ mice can support the development of human NK cells and CD56+ T cells after intrahepatic injection with hematopoietic stem cells. The human CD56^+^ cells in BALB/c Rag2^−/−^/γ_c_^−/−^ mice were able to produce IFN-γ in response to human IL-15 and polyI:C. NK cells from reconstituted Rag2^−/−^/γ_c_^−/−^ mice were also able to kill and inhibit the growth of K562 cells *in vitro* and were able to produce IFN-γ in response to stimulation with K562 cells. *In vivo*, reconstituted Rag2^−/−^/γ_c_^−/−^ mice had higher survival rates after K562 challenge compared to non-reconstituted Rag2^−/−^/γ_c_^−/−^ mice and were able to control tumor burden in various organs. Reconstituted Rag2^−/−^/γ_c_^−/−^ mice represent a model in which functional human NK and CD56+ T cells can develop from stem cells and can thus be used to study human disease in a more clinically relevant environment.

## Introduction

Mouse models are used extensively for the study of human disease in many fields including cancer. Although mice have provided valuable insights into disease initiation, progression and therapy, they do not accurately represent human immune responses and results obtained in mice are therefore difficult to extrapolate to humans. Although it is clear that humans cannot be used as subjects in many experiments, a model which better represents the human response would be a logical step forward. A humanized mouse model represents a valuable means of assessing human immune responses to diseases such as cancer.

Several immunodeficient mouse strains have been used to engraft human stem cells. NOD/Scid mice have been shown to consistently support human stem cell engraftment and have been used extensively [1]–[3]. However, NOD/Scid mice suffer from a high rate of thymomas, the presence of residual NK cells and spontaneous development of murine T and B cells. In addition, they do not develop human T or NK cells without further manipulation [4]–[6]. It is therefore difficult to use these mice in long-term immunological studies. Improved models which lack the problems of the NOD/Scid strain and support full differentiation of human stem cells have since been developed. In one model, the NOD/Scid strain was modified by backcrossing it with the common cytokine receptor gamma chain knockout mouse (γ_c_^−/−^) [7] and in a similar model, the γ_c_^−/−^ mouse was genetically crossed with the recombinase activating gene 2 knockout (Rag2^−/−^) mouse [8]. Rag2^−/−^/γ_c_^−/−^ mice are alymphoid, lacking B cells, T cells and NK cells and are excellent recipients for human cell engraftment. It is interesting to note however, that engraftment in these mice is dependent on both the age of the mice at the time of stem cell delivery and the background strain of the mice as only newborn Rag2^−/−^/γ_c_^−/−^ mice on a BALB/c background appear to support engraftment [9]. These restrictions do not seem to apply to mice with the NOD/Scid mutations which support engraftment at any age, although differentiation of T and NK cells in these mice does not occur without further manipulation. Most studies to date have focused on generating human adaptive immune responses in humanized mouse models and several successful studies have been reported. Human innate immune responses have also been examined in this model, although to a lesser extent. Functional dendritic cells have been shown to develop in the reconstituted BALB/c Rag2^−/−^/γ_c_^−/−^ mice [10]. Human NK cells have also been shown to develop [11] and in a recent study, have been shown to respond to exogenously administered human IL-15 which improved their development *in vivo* [12].

The innate immune system is the first line of defense and is crucial for containing and eliminating a wide range of tumor cells or microbial pathogens without prior exposure. Both NK and non-MHC restricted NKT cells have been shown to have innate anti-tumor activity. For example, in patients with acute myeloid leukemia (AML) undergoing allogenic stem cell transplantation, alloreactive NK cells have been shown to have antileukemic effects which resulted in improved survival of the patients [13]. Furthermore, activated human NK cells have been shown to have a potential role in treatment of AML when adoptively transferred into patients [14]. NKT cells have been shown to have non-MHC restricted innate anti-tumor activities similar to those of NK cells however these innate functions have only been examined *in vitro* [15], [16]. A possible role for NK/NKT cells in preventing the natural development of cancer has been shown in a cohort study in which low levels of cytotoxic activity of peripheral blood lymphocytes was linked to an increased risk of cancer [17]. Studies have shown that NK and NKT cell precursors exist within the human CD34^+^ hematopoietic cell population in umbilical cord blood and can differentiate into mature NK cells *in vitro* in the presence of IL-15 and cytokines such as flt3 ligand or stem cell factor [18]–[21].

Our understanding of human NK and NKT cell function has primarily been based on *in vitro* analyses, and *in vivo* models to study human NK/NKT cells are lacking. Valuable information has been gained from studying murine NK/NKT cells and it has been shown that both NK and NKT cells have potent anti-tumor activities *in vivo* [22]–[25]. However, while there are similarities between murine and human NK cells, there are also many differences which make it difficult to implement results obtained from murine studies into humans [26]. In an attempt to examine human NK cell function *in vivo*, several studies have used immunodeficient mice into which human tumor cells or pre-differentiated and activated human NK cells were injected. These studies have shown that NK cells can inhibit tumor growth and that the absence of NK cells enhances tumorigenicity [27], [28]. Although mouse models have the capacity to support NK/NKT cell activity and tumor growth, they do not develop NK/NKT cells from *in vivo* precursors and therefore may not accurately represent natural human responses. In this study, we show that human CD56+ NK and T cells develop *in vivo* from hematopoietic stem cells in BALB/c Rag2^−/−^/γ_c_^−/−^ mice injected as newborns but not C57BL/6 Rag2^−/−^/γ_c_^−/−^ mice or NOD/Scid mice. The human CD56+ cells are able to produce IFN-γ in response to polyI:C and IL-15 and cells from reconstituted mice are able to respond to the human NK-sensitive K562 erythroleukemia cell line by producing IFN-γ and inhibiting tumor growth both *in vitro* and *in vivo*.

## Materials and Methods

### Ethics

All animal experiments were approved by the Animal Research Ethic Board (AREB) of McMaster University. Use of human cord blood was approved by the Research Ethic Board (REB) of McMaster University. Human cord blood samples were obtained with parent written informed consent.

### Mice

NOD/Scid mice were purchased from The Jackson Lab (Bar Harbor, ME). C57BL/6 Rag2^−/−^/γc^−/−^ mice were purchased from Taconic (Germantown, NY). BALB/c Rag2^−/−^/γc^−/−^ mice were generously provided by M. Ito (Central Institute for Experimental Animals, Kawasaki, Japan). All mice were bred and maintained under specific pathogen-free conditions at the McMaster University Medical Center. Mouse colonies were maintained on a 12 h light/12 h dark light cycle.

### Transplantation of Human Cord Blood-Derived Stem Cells into Mice

Human cord blood samples were obtained with parent written informed consent. (Department of Labour and Delivery, McMaster University Medical Center, Hamilton, Ontario). Mononuclear cells were enriched from whole blood using HetaSep (Stem Cell Technologies, Vancouver, British Columbia). CD34^+^ cells were enriched by removing lineage committed cells using a cocktail of antibodies with the RosetteSep system according to the manufacturer's instructions (Stem Cell Technologies, Vancouver, BC). Injected cells were 70–80% CD34^+^ and no lineage positive cells remained. Samples were frozen immediately until use.

Mice were injected either at 6–8 weeks of age or on the day of birth. Adult NOD/Scid mice were irradiated with 3.5 Gy and adult Rag2^−/−^/γc^−/−^ mice were irradiated with 5–6 Gy. Both strains were injected with 1–3×10^6^ CD34-enriched cells into the tail vein. Newborn mice were irradiated twice in a 3–4 hour interval. Newborn NOD/Scid mice received 1.5×1.5 Gy and newborn Rag2^−/−^/γc^−/−^ mice recieved 3×3 Gy. After the second dose of irradiation, newborn mice were injected intrahepatically with approximately 1–2×10^6^ CD34-enriched cells in 30 ul PBS using a 30-guage needle. Control mice were irradiated using the same protocol but did not receive any human cells. Mice were weaned at 3 weeks of age.

### Analysis of Human Cell Engraftment

At 12–20 weeks post-transplantation, mice were euthanized and examined for the presence human lymphocytes. Single cell suspensions were made from bone marrow, spleen, lymph nodes, thymus and blood. Spleens, lymph nodes and thymus were passed through a 40 um filter and red blood cells were removed with ACK lysis buffer.

Cells were stained for FACS with antibodies against human CD45 PE (HI30), CD56 PE (B159), (NCAM16.2), CD3 FITC (HIT3a), CD16 PE-Cy5 (3G8) (BD Pharmingen, San Diego, California), CD94 APC (DX22), KIR3DL1 Alexa Fluor 700 (DX9), NKp30 APC (P30-15), NKp44 PE (P44-8), NKG2D PE (1D11), KIR2DL2/L3 FITC (DX27) (Biolegend, San Diego, CA) and NKp46 FITC (195314) (R&D Systems, Minneapolis, MN). Flow cytometric data was collected using a FACScan, LSR II or FACS Canto (Becton Dickinson) and was analyzed using FlowJo software. For data collected using the FACScan, compensation was done manually using single-stained cells. For data collected using the LSR II and FACS Canto, compensation was done with FACS Diva software using single-stained compensation beads.

### NK Cell Stimulation and IFN-γ measurement

Naïve reconstituted mice were euthanized and spleen and mesenteric lymph nodes were removed. Single cell suspensions from these organs were incubated with K562 cells for 72 hours. In a separate experiment, 5×10^5^ spleen and lymph node cells were cultured with human IL-2 (400 U/ml; R&D Systems, Minneapolis, MN) and either recombinant human IL-15 (200 ng/ml; R&D Systems, Minneapolis, MN) or polyI:C (25 µg/ml, Sigma, Oakville, ON) for 72 hours. IFN-γ production by NK cells was measured in both experiments by ELISA and/or by FACS using CD56 surface staining and intracellular cytokine staining. For intracellular cytokine staining, GolgiPlug (1 ul/ml; BD Biosciences, San Diego, CA) was added to the cultures for the last 6 hours.

### CD107a degranulation assay

Cells from the spleen and thymus of reconstituted mice were used as effectors in a CD107a assay. Cells were incubated with K562 target cells or left unstimulated to detect spontaneous degranulation. Effectors and targets were plated at an E∶T ratio of 2∶1 and incubated for 4 hours at 37°C and 5% CO_2_. Anti-human CD107a mAb FITC (H4A3) (Biolegend, San Diego, CA) was added to each well at the start of the incubation (20 ul/well). After 1 hour, 4 ul of BD GolgiStop (BD Biosciences, San Diego, CA) was added for every 6 ml of cell suspension. After the incubation, cells were stained with antibodies against human CD45 (BD Pharmingen, San Diego, CA), CD3 PerCP-Cy5.5 (HIT3a) and CD56 PE-Cy7 (HCD56) (Biolegend, San Diego, CA) for flow cytometric analysis. Data was collected using a FACS Canto (Becton Dickinson) and analyzed using FlowJo software.

### In vitro K562 cell growth inhibition measurement

Spleen and lymph node cells from naïve reconstituted Rag2^−/−^/γc^−/−^ mice and non-reconstituted Rag2^−/−^/γc^−/−^ and BALB/c controls were isolated and cultured in 10% RPMI with K562 cells at a 50∶1 effector to target ratio. After 60 hours, human IL-2 (400 U/ml) and IL-15 (100 ng/ml) were added to the cultures for an additional 10 hours. The number of surviving cells was determined by adding 1×10^5^ FITC-labeled beads to each tube and counting the number of K562 cells in the culture by FACS. The following equation was used: number of added beads (1×10^5^)×number of counted targets (K562)/number of counted beads. Since the cells were cultured for 72 hours, specific growth inhibition of K562 cells was measured instead of specific lysis. Calculation of % growth inhibition was adapted from a recent study [29] and was calculated as (number of surviving K562_spontaneous_–number of surviving targets_experimental_)/(number of surviving targets_spontaneous_)×100.

### K562 Tumour Injection and Monitoring

At 10–12 weeks post-transplantation, 5×10^5^ K562 cells were injected into the tail vein of mice. After injection mice were weighed and monitored daily for sickness and tumor formation. Mice were monitored for up to 90 days and were euthanized when approximately 20% of total body weight was lost or when tumors were large enough to impair eating or walking. In a separate experiment, reconstituted and non-reconstituted mice were euthanized 40 days after challenge and bone marrow, spleen, liver and blood were removed. Single cell suspensions were stained for FACS with glyA PE (GA-R2) (BD biosciences) to detect tumour cells.

### CD56 depletion

Anti-human CD56 IgM antibodies were prepared from the supernatant of HNK-1 cells (ATCC) Cells were cultured according to ATCC recommendation for propagation and then cultured in serum free media for antibody production. Cells were pelleted and supernatants were then centrifuged to remove any cell debris. The IgM in the supernatant was then precipitated with saturated ammonium sulfate to reach a 40% solution and then stored at 4°C overnight. The protein precipitate was then centrifuged and dialyzed in several changes of PBS. The purity of IgM were tested with SDS-page gel and the IgM concentration was determined by both ELISA and Bradford assay. The purified anti-CD56 antibodies were injected into reconstituted and non-reconstituted mice (0.2 mg/mouse) 2 days before administration of K562 cells and every 2–3 days after K562 cell injection. Delivery of K562 cells and monitoring of mice was performed as described in the previous section. The anti-CD56 antibody delivery was maintained until endpoint when the mice succumbed to the tumor burden. CD56-depleted mice were examined for the presence of CD56^+^ cells by FACS after the first 2 antibody injections and at endpoint to ensure the depletion was maintained. CD56 staining was performed using 2 different antibody clones. In addition, cells were also stained for CD16.

### Statistics

Survival data was analyzed using the chi-squared test. Differences in IFN-γ levels, growth inhibition and % dead cells between reconstituted and non-reconstituted mice as well as differences in degranulation between co-culture with and without K562 cells were evaluated using Mann-Whitney Rank Sum test.

## Results

### Reconstitution and development of CD56^+^ NK and T cells in immunodeficient mouse strains

**Figure 1:**
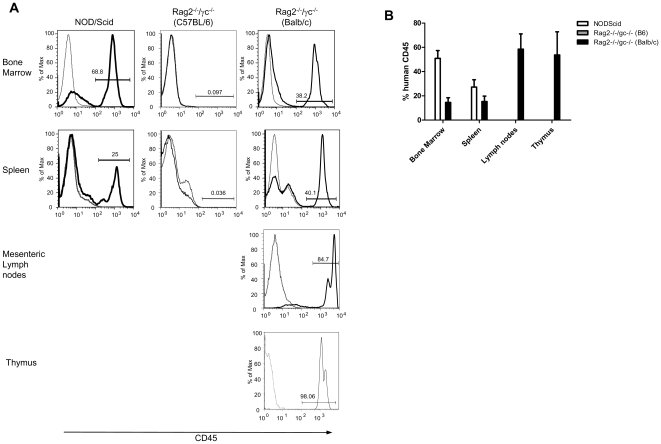
Analysis of reconstitution in immunodeficient mice. Newborn NOD/Scid, C57BL/6 Rag2^−/−^/γ_c_^−/−^ and BALB/c Rag2^−/−^/γ_c_^−/−^ mice were examined 12 weeks after injection with human cord blood derived hematopoietic stem cells. (A) Bone marrow, spleen, lymph nodes and thymus were examined by FACS for the presence of human CD45^+^ cells. For bone marrow and spleen n = 20 mice, for lymph nodes n = 10 mice, for thymus n = 5 mice. Lightweight line represents unreconstituted BALB/c Rag2^−/−^/γ_c_^−/−^ mouse, dark line represents reconstituted BALB/c Rag2^−/−^/γ_c_^−/−^ mouse. Isotype controls were also negative (not shown) (B) Summary graph of human CD45 reconstitution in mouse strains and tissues The y axis, in figure 1b, is of the irrelevant channel.

At 12–14 weeks post-transplantation, single cell suspensions from bone marrow, spleens and lymph nodes were examined for the presence of human lymphocytes. Human CD45^+^ cells were observed in the bone marrow and spleen of all NOD/Scid and BALB/c Rag2^−/−^/γc^−/−^ mice that had received grafts as newborns (fig. 1A, B). Similar levels of reconstitution were detected in NOD/Scid mice that received grafts as adults but no CD45^+^ cells were present in adult BALB/c Rag2^−/−^/γc^−/−^ recipient mice (data not shown). No reconstitution was detected in newborn or adult C57BL/6 Rag2^−/−^/γc^−/−^ recipient mice. Only BALB/c Rag2^−/−^/γc^−/−^ mice that received grafts as newborns developed detectable lymph nodes which were almost entirely made up of human CD45^+^ cells (fig. 1A). As expected, none of the non-reconstituted mice developed lymph nodes.

**Figure 2:**
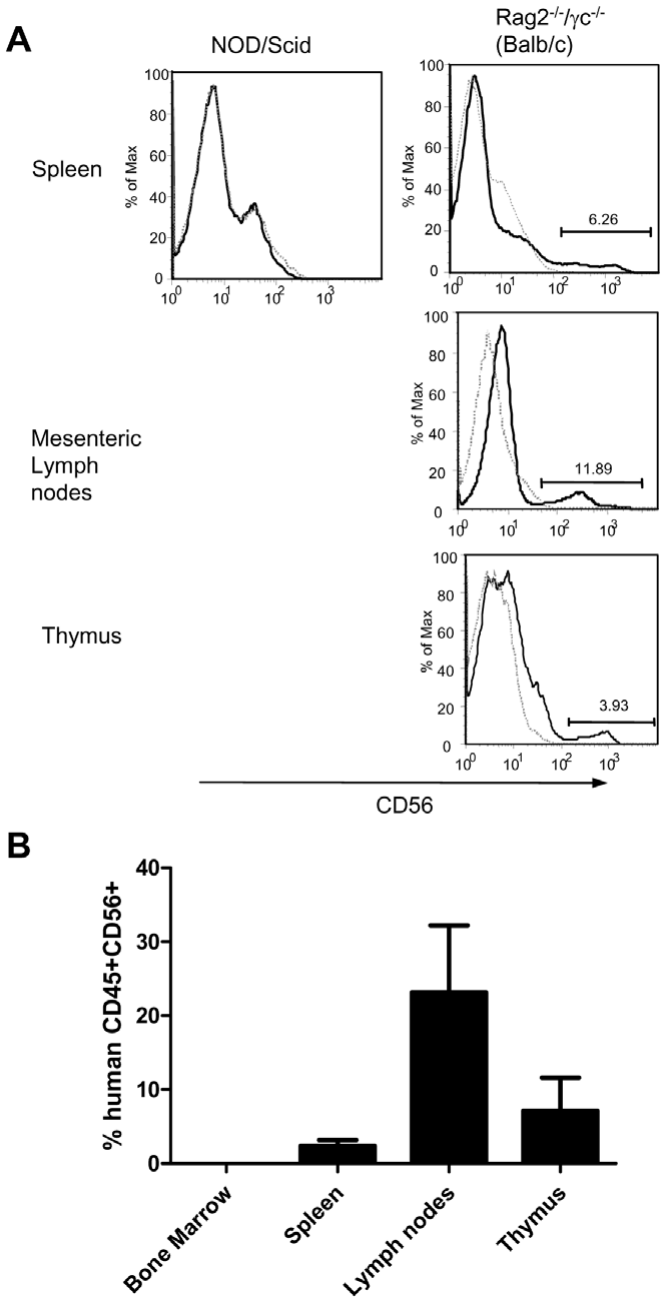
Analysis of NK cell reconstitution in immunodeficient mice. Newborn NOD/Scid, C57BL/6 Rag2^−/−^/γ_c_^−/−^ and BALB/c Rag2^−/−^/γ_c_^−/−^ mice were examined 12 weeks after injection with human cord blood derived hematopoietic stem cells. For bone marrow and spleen n = 20 mice, for lymph nodes n = 10 mice, for thymus n = 5 mice. Lightweight line represents unreconstituted BALB/c Rag2^−/−^/γ_c_^−/−^ mouse, dark line represents reconstituted BALB/c Rag2^−/−^/γ_c_^−/−^ mouse. Isotype controls were also negative (not shown) (A) Spleen, lymph nodes and thymus were also examined for the presence of human CD56^+^ cells. (B) Summary of CD45+CD56+ cells in tissues of BALB/c Rag2^−/−^/γ_c_^−/−^ mice. For all tissues n = 8 mice.

Since we were interested in the innate immune response, we next determined whether human CD56^+^ cells developed in the reconstituted mice. Neither newborn nor adult NOD/Scid recipient mice developed any CD56^+^ cells in the spleen (fig. 2A). BALB/c Rag2^−/−^/γc^−/−^ mice developed CD56^+^ cells in the spleen, lymph nodes and thymus which were present at variable levels in each mouse. We detected between 0.5% and 8% human CD56^+^ cells in the spleen, between 8% and 40% in the lymph nodes and between 0.5% and 7% in the thymus (fig. 2A, B). These results indicate that only the newborn BALB/c Rag2^−/−^/γc^−/−^ model has the potential to be used for innate immune and NK cell experiments and is therefore the only strain used in the remainder of this study.

### Distribution and characterization of human CD56^+^ cells in BALB/c Rag2^−/−^/γc^−/−^ mice

**Figure 3:**
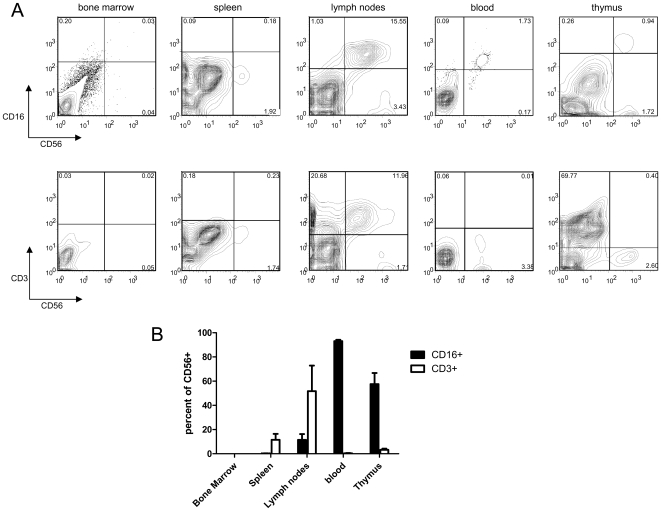
Development of NK and NKT cells in reconstituted BALB/c Rag2^−/−^/γ_c_^−/−^ mice. Cells from bone marrow, spleen, mesenteric lymph nodes, blood and thymus of reconstituted mice were collected 12 weeks after injection of human hematopoietic stem cells and were stained for human CD56 and either CD16 or CD3. (A) Represenative FACS plots. All plots were gated on the lymphocyte population. All the gates in this figure were set based on isotype controls which were run at the same day with the same samples. Isotype controls were negative for all antibodies (supplemental data, figure 1). (B) Summary of staining from each tissue. Bars represent the mean percentage of CD56+ cells that are either CD3+ or CD16+ (±SEM). Data is representative of 4 mice.

We next examined the tissue distribution of the CD56^+^ cells in reconstituted mice as well as the presence of CD3 and CD16 on these cells. As shown in Figure 2, no CD56^+^ cells were detected in the bone marrow. As mentioned above, reconstitution of CD56^+^ cells was quite variable in the spleen however, the majority of the CD56 positive cells that developed were negative for both CD16 and CD3 (fig. 3). Variability in the proportion of CD56^+^ cells was also observed in the mesenteric lymph nodes however, a much larger percentage of the cells in the lymph node expressed CD56 (fig. 2). Of the CD56^+^ cells, most expressed CD16 however there was a population that was also CD16^−^. In addition, CD3 was detected on the majority of CD56^+^ cells in the lymph nodes (fig. 3) indicating that many of the cells displayed an NK and T cell phenotype. In the blood, cells were CD56^+^CD16^+^ and CD56^+^CD3^−^ indicating a lack of T cells and in the thymus CD56^+^ cells were either CD16 positive or negative (fig. 3). Each time the cells from a tissue were run through the flow cytometer, isotype controls were also included (Figure S1).

**Figure 4:**
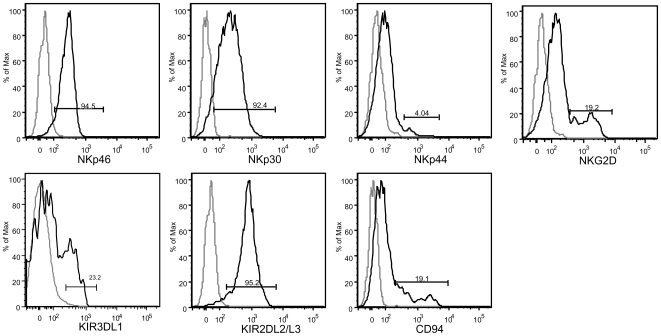
Expression of human NK cell markers on cells from reconstituted BALB/c Rag2^−/−^/γ_c_^−/−^ mice. Cells from the thymus of reconstituted mice were isolated and stained for FACS analysis. Cells were stained with antibodies against NKp46, NKp30, NKp44, NKG2D, KIR3DL1, KIR2DL2/L3 and CD94 (dark line). All plots are gated on CD45^+^CD56^+^CD3^−^ cells. Light gray line represents staining with isotype control antibody. Figures are representative of 4 mice.

In order to further characterize the human NK cells in reconstituted mice, CD56^+^CD3^−^ cells were stained with several NK cell inhibitory and activation markers (fig. 4). Figure 3 shows staining on cells from the thymus as this tissue had the highest number of CD56^+^CD3^+^ cells. Expression levels of the natural cytotoxicity receptors (NCR) NKp46 and NKp30 as well as KIRs was variable between mice however, expression was consistently positive indicating a mature phenotype. Approximately 20% of NK cells expressed the activating receptor NKG2D and a similar percentage expressed the inhibitory receptor CD94. NKp44 was present on a small population of NK cells (fig. 4).

### Presence of functional human CD56^+^ NK and T cells in BALB/c Rag2^−/−^/γc^−/−^ mice

**Figure 5:**
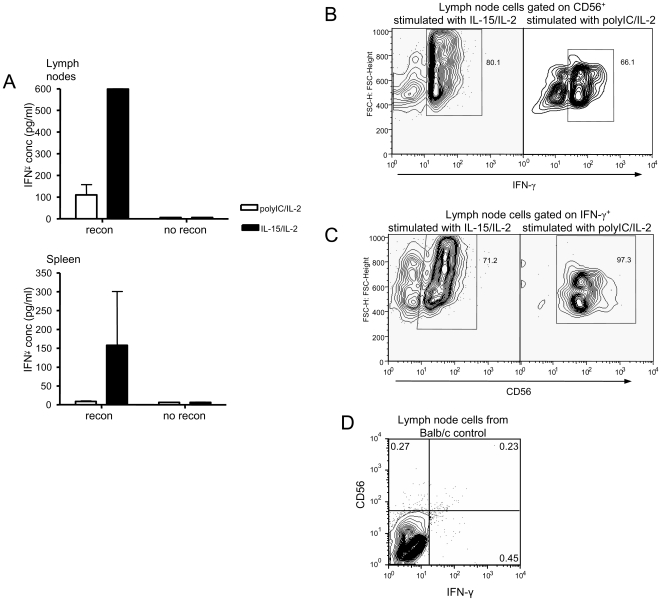
Human IFN-γ production by NK cells from reconstituted BALB/c Rag2^−/−^/γ_c_^−/−^ mice. Mesenteric lymph node or spleen cells were stimulated with human IL-15/IL-2 or polyI:C/IL-2 for 48 hours. (A) Human IFN-γ was detected in the supernatants of both lymph node (top) and spleen (bottom) cultures by ELISA. n = 4 mice for each tissue and treatment (B) Cells from lymph node cultures were stained for FACS with human CD56 and IFN-γ. Cells were gated on CD56^+^ and analyzed for production of IFN-γ. (C) In a separate analysis, lymph node cells were gated on IFN-γ^+^ and stained with anti-human CD56. (D) Lymph node cells from Balb/c mice were stimulated as above and stained with CD56 and IFN-γ as controls to ensure no non-specific staining occurred. Figures B–C are representative of 4 mice. Figure D is representative of 5 mice.

After establishing that human CD56^+^ NK and T cells can develop in newborn BALB/c Rag2^−/−^/γc^−/−^ mice, we next determined whether the cells are functional. Spleen and lymph node cells from reconstituted mice and non-reconstituted Rag2^−/−^/γc^−/−^ or BALB/c control mice were stimulated with human IL-2 and either IL-15 or polyI:C. After 72 hours, human IFN-γ was detected in all of the reconstituted culture supernatants by ELISA (fig. 5A). In order to confirm that the IFN-γ was being produced by human CD56^+^ cells, intracellular cytokine staining (ICCS) was performed. In the lymph nodes, up to 80% of the CD56^+^ cells from reconstituted mice produced IFN-γ in response to human IL-15/IL-2 and up to 66% of CD56^+^ cells produced IFN-γ in response to polyI:C (fig. 5B). To further confirm this observation, we determined that the majority of the IFN-γ positive cells in the cultures were CD56^+^ (fig. 5C). These observations further indicate that *in vivo*, early IFN- γ is likely produced by CD56^+^ cells.

### Ability of human cells to kill and inhibit K562 cell growth in vitro

After determining that the human CD56^+^ cells from reconstituted Rag2^−/−^/γc^−/−^ mice could produce IFN-γ, we examined another fundamental and unique function of human NK cells; their ability to kill or inhibit the growth of K562 cells.

**Figure 6:**
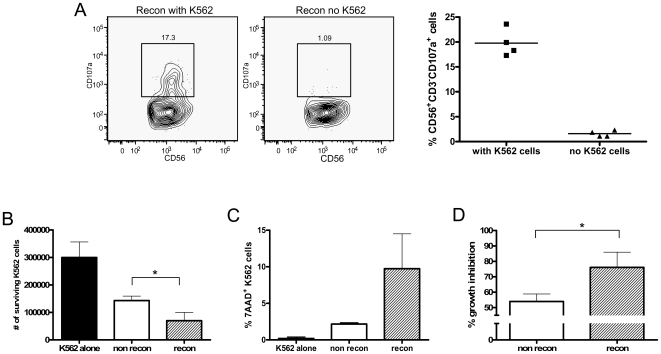
NK cell degranulation and inhibition of K562 cell growth by spleen cells. (A) K562 cells were cultured with thymus cells from reconstituted mice for 4 hours. Cells were stained with CD56, CD3 and CD107a in order to measure degranulation of NK cells. Contour plots show representative CD107a staining on CD56^+^CD3^−^ cells cultured with or without K562 cells. Bar graph shows significantly greater expression of CD107a on cells cultured with K562 stimulation compared to cells cultured in the absence of K562 stimulation (p<0.005). n = 4 mice (B–D) K562 cells were cultured with spleen cells from reconstituted mice, non-reconstituted mice or alone for 72 hours and then harvested and stained for analysis by FACS. (B) The total number of surviving K562 cells was determined as described in materials and methods. (C) The percentage of dead K562 cells in the cultures was determined by staining with 7AAD. (D) % growth inhibition of K562 cells was calculated as described in materials and methods. For all experiments in B–D, reconstituted n = 3, non-reconstituted n = 6. Bars represent mean±SEM. * indicates significant difference between the reconstituted and non-reconstituted groups (p<0.05).

A 4-hour CD107a degranulation assay was performed using thymus cells from reconstituted mice cultured with NK-sensitive K562 cells. There was a significant difference in the percentage of NK cells that expressed CD107a after culture with or without K562 cells (fig. 6A, p<0.005). CD107a expression by human CD56^+^CD3^−^ cells was detected in approximately 20% of cells that were cultured with K562 cells compared to just 1% of cells cultured in the absence of K562 cells (fig. 6A), indicating that human NK cell are able to degranulate in response to K562 target cells. No increase in CD107a expression was observed on CD56^+^CD3^+^ cells (data not shown).

In a long-term *in vitro* experiment, spleen and lymph node cells from reconstituted and non-reconstituted mice were isolated and cultured with K562 cells for 72 hours. The total number of K562 cells in the culture after this time was measured by FACS and compared between groups. K562 cells cultured alone were used as an additional control. As shown in figure 6B, the number of K562 cells cultured in the presence of spleen cells from reconstituted mice was significantly less than that measured from cultures with cells from non-reconstituted mice or no exogenous cells (*p*<0.05). A decrease in the number of surviving K562 cells was also observed in the lymph node cultures from reconstituted mice although the results were not as dramatic as seen in the spleen (data not shown). In addition, compared to cultures with non-reconstituted cells, approximately four times more K562 cells were dead in the reconstituted spleen cell cultures (fig. 6C). These results indicate that the human cells from reconstituted mice have a significant effect on the growth of K562 cells *in vitro*.

We then quantified the effect of human CD56^+^ cells on K562 cells, by calculating the percentage of K562 growth inhibition when cultured with spleen cells compared to uninhibited growth. As shown in figure 5D, spleen cells from non-reconstituted mice inhibited growth by only 47% over 72 hours whereas growth inhibition by spleen cells from reconstituted mice was significantly higher at an average of 80% with cells from some mice causing up to 96% growth inhibition (*p*<0.05). It is thus clear that the human CD56^+^ cells from reconstituted mice can almost completely block the expansion of K562 cells *in vitro*.

In order to further demonstrate that the human CD56^+^ cells are active in response to K562 cells, human IFN-γ production by CD56^+^ cells was measured by ICCS. Lymph node cells from reconstituted mice were isolated and cultured with K562 cells for 72 hours. Cells were then collected and stained for the expression of human CD56 and IFN-γ. Almost all of the CD56^+^ cells were active in response to the K562 cells and expressed IFN-γ. In addition, the IFN-γ was being produced exclusively by CD56^+^ cells as over 99% of the IFN-γ^+^ cells in the culture were CD56^+^ (data not shown).

### Survival of reconstituted Rag2^−/−^/γc^−/−^ mice after K562 cell injection

**Figure 7:**
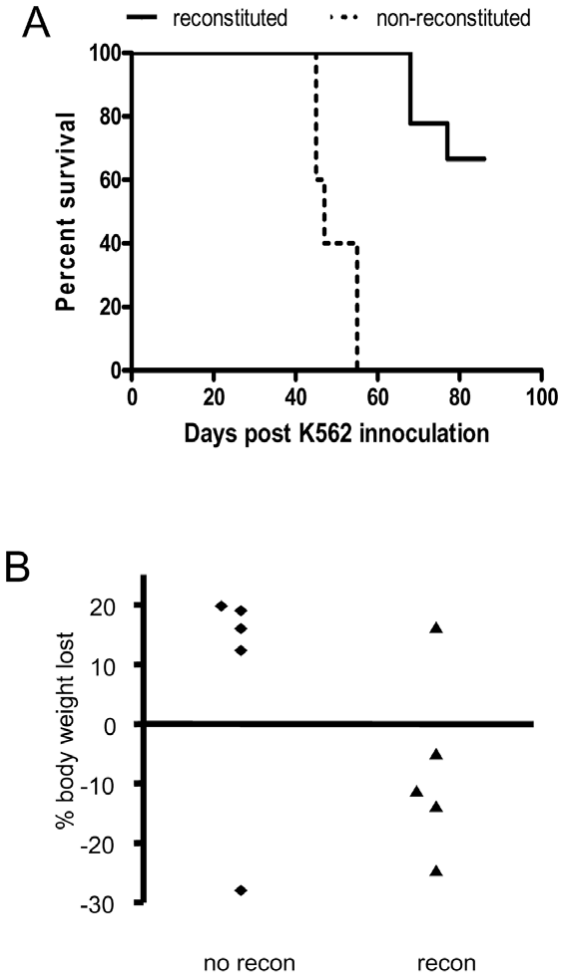
Survival and body weight after K562 challenge. (A) Mice were challenged i.v. with K562 cells and monitored for survival. (n = 9 reconstituted mice, n = 6 non-reconstituted mice). (B) After K562 challenge, mice were also monitored daily for changes in body weight (n = 5).

After determining that the human NK cells from reconstituted Rag2^−/−^/γ_c_^−/−^ mice could inhibit growth and produce IFN-γ in response to K562 cells *in vitro*, we examined whether these cells could respond to tumor cells *in vivo*. In two separate experiments, 9 reconstituted and 6 non-reconstituted Rag2^−/−^/γ_c_^−/−^ mice were injected i.v. with K562 cells and were monitored for loss of body weight and tumor formation. Both groups were irradiated as newborns. As shown in figure 7, it is clear that the reconstituted mice were able to survive the tumor challenge significantly better than the non-reconstituted mice (p<.0001). Further demonstrating their ability to defend against tumor challenge, reconstituted mice showed an overall gain in total body weight whereas non-reconstituted Rag2^−/−^/γc^−/−^ mice lost approximately 20% of their total body weight (fig. 7). Reconstituted and non-reconstituted mice that were not challenged with K562 cells both survived and gained body weight equally up to the 90 day experimental endpoint (data not shown). The majority of the reconstituted mice did not develop any detectable tumors and lived beyond the 90 day experimental endpoint (Figure S2). Those that did develop tumors were not euthanized until 68 days after tumor challenge compared to the non-reconstituted mice which developed large tumors and were euthanized between 45 and 55 days after K562 cell injection. These results show that human CD56^+^ cells in reconstituted mice are able to respond to K562 tumor cell challenge and likely contribute to the protective immune responses.

### Tumor burden and IFN-γ production in K562-challenged Rag2^−/−^/γc^−/−^ mice

**Figure 8:**
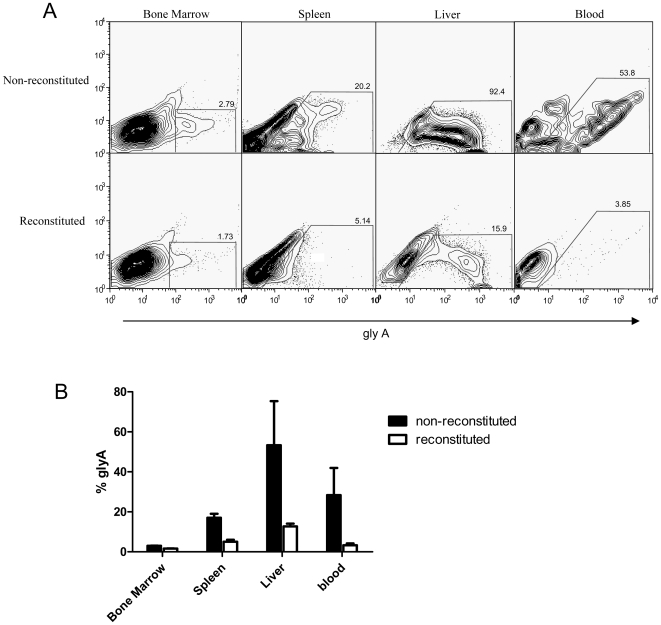
Tumour burden and IFN-γ production in K562-challenged Rag2^−/−^/γc^−/−^ mice. Reconstituted and non-reconstituted mice were challenged i.v. with K562 cells. Various organs were removed from mice after 40 days and stained for glyA expressed by K562 cells. (A) Representative figures showing glyA expression in different tissues. The y axis is an irrelevant channel. (B) Graph summarizing data from all mice analyzed. N = 3 resonstituted mice and n = 4 non-reconstituted mice.

In order to determine the extent to which human cells were able to inhibit K562 cell growth *in vivo*, tumor burden was measured in both reconstituted and non-reconstituted Rag2^−/−^/γc^−/−^ mice. At 40 days after K562 cell injection, reconstituted and non-reconstituted mice were euthanized and analyzed for the presence of tumor cells. Mice that reached endpoint developed large solid tumors in the abdomen, primarily encompassing the kidneys and liver as well as in the jaw. Circulating tumor cells were also detected. In order to determine the significance of the tumor burden in the reconstituted and non-reconstituted mice, single cell suspensions of bone marrow, spleen, liver and blood were prepared and stained with anti-human glycophorin A which is expressed by K562 cells. All non-reconstituted organs examined contained significantly higher percentages of K562 cells than organs from reconstituted mice. As shown in figure 8, most K562 cells engrafted into the liver however, K562 cells were also detected in the bone marrow, spleen and circulation. Cultured K562 cells before injection stained positive for glycophorin A as did cells from solid tumors removed from the abdomen of non-reconstituted mice (not shown). Reconstituted mice that survived up to 90 days after K562 cell injection were able to completely clear the tumor cells as none were detected by FACS staining (Figure S2). This is a clear illustration of the degree of protection offered by the human cells in reconstituted mice. In addition, spleen cells from reconstituted mice that were challenged with K562 produced 12-fold more human IFN-γ (as measure by ELISA) than spleen cells from naïve reconstituted mice when stimulated for 48 hours with polyI:C and 3-fold more when stimulated with IL-15 (data not shown).

### CD56 depletion and K562 tumor challenge

**Figure 9:**
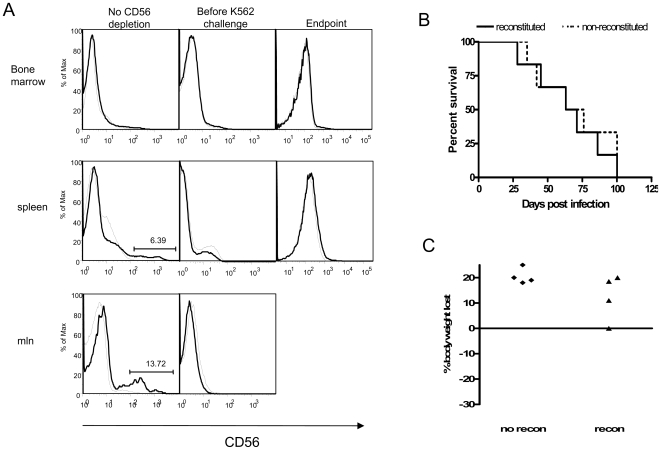
CD56 depletion of reconstituted Rag2^−/−^/γc^−/−^ mice and K562 challenge. 10 weeks after injection of human hematopoietic stem cells, reconstituted mice were injected with anti-CD56 antibodies i.p and challenged i.v. with K562 cells. Injections were administered 2 consecutive days before administration of K562 cells and every 3 or 4 days after K562 cell delivery. Mice were examined for the presence of CD56+ cells before K562 cell delivery and at endpoint to ensure the depletion was maintained. (A) Lightweight line represents isotype control, dark line represents stain with human anti-CD56 antibody. (B) Survival rates in the CD56-depleted reconstituted mice and non-reconstituted mice (n = 6). (C) After K562 challenge, mice were also monitored daily for changes in body weight. (n = 4).

In order to confirm the role of CD56^+^ cells in tumor protection, reconstituted and non-reconstituted mice were injected with anti-CD56 antibodies to deplete NK cells. Mice were then injected i.v. with 5×10^5^ K562 cells and monitored for survival. As shown in figure 9A, reconstituted mice were completely depleted of CD56^+^ cells both before K562 cell injection and at endpoint indicating that the depletion was maintained throughout the experiment. Reconstituted mice that were depleted of CD56^+^ cells had survival rates equal to that of non-reconstituted mice (fig. 9B) and the body weight of all mice dropped equally (fig. 9C) indicating that there was no difference in protection between the reconstituted CD56-depleted and the non-reconstituted mice. Reconstituted mice that were injected with K562 cells but not treated with anti-CD56 all survived until the experimental endpoint at day 100 post K562 innoculation (n = 4; data not shown). These results indicate that human CD56^+^ cells play a significant role in the protection that was observed in reconstituted non-CD56-depleted mice (fig. 7).

## Discussion

NK cells are a vital component of the innate immune system and have long been implicated in the detection and elimination of tumor cells [30]. In this study, we examined whether immunodeficient mice could support the development of human NK and NKT like (CD56^+^/CD3^+^) cells in various tissues and whether these cells could effectively target human K562 tumor cells.

We examined several immunodeficient mouse models and found, as previously reported, that while engraftment in NOD/Scid mice is not dependent on age at the time of stem cell delivery, Rag2^−/−^/γ_c_^−/−^ mice are only able to support high levels of engraftment when stem cells are delivered at birth [9], [31]. In addition, the background strain of the Rag2^−/−^/γ_c_^−/−^ mice appears to play a crucial role in graft acceptance as C57BL/6 Rag2^−/−^/γ_c_^−/−^ mice (at any age) do not support engraftment whereas newborn BALB/c Rag2^−/−^/γ_c_^−/−^ mice are able to support full differentiation of human immune cells [11], [16]. Although this observation has not yet been explained, it is possible that differences in growth factors exist within the bone marrow environment of the two strains which influence engraftment. It is also possible that known differences in immune responses between BALB/c and C56BL/6 mice could affect engraftment. The focus of this study was on human NK cells and it was therefore crucial to determine which mouse strain would best support NK cell development. We have shown here that reconstituted NOD/Scid mice, regardless of age, are devoid of human CD56^+^ cells while reconstituted newborn Rag2^−/−^/γ_c_^−/−^ mice develop them in the spleen, lymph nodes and thymus. The lack of CD56^+^ cell development in the NOD/Scid mice is likely due to interference by residual murine NK cells which are not present in Rag2^−/−^/γ_c_^−/−^ mice. We therefore determined that the newborn BALB/c Rag2^−/−^/γ_c_^−/−^ mice were the most suitable model for examining the role of human CD56^+^ cells in innate tumor immunity.

It is well known that NK cells and possibly NKT cells play a crucial role in the innate immune response. They are highly responsive to a variety of cytokines including IL-2, IL-12 and IL-15 and following interaction of NK cell surface receptors with cognate ligands, they mediate cytotoxicity via perforin/granzyme granule-mediated exocytosis and produce several cytokines including IFN-γ [23], [32]. Previous studies have shown that administration of exogenous cytokines, such as IL-15, is required for the development of mature NK cells *in vitro* and in immunodeficient mice [20], [33]–[35]. A recent study by Huntington et.al., [12] examined the role of IL-15 in regulating the development of human NK cells in BALB/c Rag2^−/−^/γ_c_^−/−^ mice. They reconstituted the mice with CD34+ human fetal liver cells and showed that exogenous delivery of human IL-15 plus IL-15 receptor agonists enhanced the development, differentiation and proliferation of NK cells [12]. In the present study, we have shown that CD56^+^ NK and T cells develop naturally in BALB/c Rag2^−/−^/γ_c_^−/−^ mice reconstituted with lineage-depleted human umbilical cord blood cells, without the need for further manipulation. The levels of NK cells observed in the present study were generally higher than those observed by Huntington et.al. which may be due to differences in the source of the human cells, the number of cells delivered or the method used to enrich hematopoietic stem cells. We have used negative selection which results in the enrichment of CD34+ as well as other possible HSCs. The distribution of the human CD56^+^ cells in the mice was interesting as CD56^+^/CD3^+^ cells were mainly detected in the lymph nodes of reconstituted mice and CD56^+^/CD3^−^ cells were detected in all tissues except bone marrow. Further examination of CD56^+^CD3^−^ NK cells revealed that several human NK markers are expressed including the natural cytotoxicity receptors (NKp46, NKp30 and NKp44), 2 KIRs, NKG2D and CD94. These observations indicate that the NK cells that develop in the BALB/c Rag2^−/−^/γ_c_^−/−^ mice are similar to those that develop naturally in humans as they express many common human NK cell markers and also behave normally by producing human IFN-γ in response to stimulation with human IL-15 as well as the classic NK-cell activator polyI:C.

The expression of CD94 and KIRs on human NK cells is important as interactions between these inhibitory receptors and self MHC class I molecules prevent killing of ‘self’ cells, whereas lack of MHC class I expression on a cell induces an NK cell response [36]–[38]. In addition, NK cell inhibitory receptors may play a role in the acquisition of functionality during NK cell development. ‘Licensing’ of NK cells is thought to involve interactions between inhibitory receptors (such as CD94, NKG2A/B/C and KIRs) and MHC class I ligands which allow NK cells to become fully functional. If this interaction does not occur, as observed in MHC class I-deficient mice, NK cells become hyporesponsive even when stimulated through activating receptors [39]–[41]. Unlicensed NK cells may still have the capacity to become functional if they are sufficiently stimulated by polyI:C, cytokines or inflammation [40], [42]. In the BALB/c Rag2^−/−^/γ_c_^−/−^ model, human NK cells are able to kill K562 target cells without prior stimulation suggesting that some form of ‘licensing’ has occurred. It is possible that human NK cell receptors may have some cross-reactivity with murine MHC class I molecules or that NK cells may be licensed by other developing human cells expressing MHC class I. It is also possible that unlicensed human NK cells become activated by cytokines present in the murine microenvironment.

There is a growing body of evidence that supports a role for NK cells in anti-tumor immunity. Much of this evidence comes from studies using mouse models in which NK cells have been depleted or strains that are deficient in NK cell development [27], [43], [44]. The role of NKT cells in anti-tumor responses is less clear however, there is evidence that they participate in tumor immunosurveillance and exert anti-tumor responses [24], [45]–[48]. In humans, *in vivo* evidence of NK/NKT-mediated tumor inhibition is lacking due to the ethical and practical restrictions on using human subjects and the absence of a clinically relevant model. In the present study, we have shown that NK cells from reconstituted BALB/cRag2^−/−^/γc^−/−^ mice could inhibit the growth of K562 leukemia cells *in vitro* compared to non-reconstituted mice. In addition, NK cells were able to kill K562 cells as well as produce IFN-γ in response to K562 stimulation *in vitro*. We have also shown that reconstituted BALB/c Rag2^−/−^/γc^−/−^ mice were protected against intravenous K562 tumor challenge compared to non-reconstituted mice. Tumor burden was much higher in non-reconstituted than in reconstituted mice examined at the same time point after K562 cell injection. This may be explained, in part, by the observation that spleen cells from reconstituted, K562-challenged mice produced more human IFN-γ than cells from reconstituted, non-challenged mice. Finally, the reconstituted mice that survived had no detectable K562 cells in any organs upon termination of the experiment at 90 days post tumor injection indicating that they completely cleared the tumor cells (Figure S2). To further support the role of NK/T cells in the anti-K562 response, reconstituted mice were depleted of CD56^+^ cells and challenged with K562 cells. The CD56-depleted mice did not survive the challenge and reached endpoint at the same time as the control, non-reconstituted mice. It is thus apparent that human NK/T cells which have developed in reconstituted mice have potent anti-tumor effects *in vivo*.

This humanized mouse model has many potential applications in the field of tumor immunity and therapy. It is an appropriate model in which to study innate immune surveillance of human tumor cells and the methods by which human immune cells naturally detect and control tumor growth. In addition, it is a clinically relevant model for examining potential NK/T cell-based anti-tumor therapies. The K562 cells used in this study are uniquely sensitive to NK cells and therefore may not be representative of most human cancers. It will be important in future studies to examine the NK cell response to more relevant tumour challenges including primary tumour cells. With the growing body of evidence supporting the anti-tumor effects of NK and NKT cells, it would be beneficial to examine the effects of exogenously administered NK activators such as IL-15 or toll-like receptor ligands on tumor prevention and treatment. Because of ethical concerns and restrictions regarding the use of humans in clinical studies, humanized mice may represent the most relevant pre-clinical model to evaluate human therapies as well as a model in which to study basic human immune functions.

## Supporting Information

Figure S1 Isotype control for Figure 3
(1.16 MB TIF)

Figure S2 (A) Reconstituted mice that survived K562 challenge completely cleared the tumor cells when examined 90 days after challenge. Lightweight line represents BALB/c Rag2−/−/gc−/− mouse not injected with K562 cells. (B) Cultured K562 cells and cells taken from solid tumors at end point were also stained for glyA. Lightweight line represents isotype control.
(0.45 MB TIF)

## References

[1] Hesselton RM, Greiner DL, Mordes JP, Rajan TV, Sullivan JL (1995). High levels of human peripheral blood mononuclear cell engraftment and enhanced susceptibility to human immunodeficiency virus type 1 infection in NOD/LtSz-scid/scid mice.. J Infect Dis.

[2] Yoshino H, Ueda T, Kawahata M, Kobayashi K, Ebihara Y (2000). Natural killer cell depletion by anti-asialo GM1 antiserum treatment enhances human hematopoietic stem cell engraftment in NOD/Shi-scid mice.. Bone Marrow Transplant.

[3] Ueda T, Tsuji K, Yoshino H, Ebihara Y, Yagasaki H (2000). Expansion of human NOD/SCID-repopulating cells by stem cell factor, Flk2/Flt3 ligand, thrombopoietin, IL-6, and soluble IL-6 receptor.. J Clin Invest.

[4] Cashman JD, Lapidot T, Wang JC, Doedens M, Shultz LD (1997). Kinetic evidence of the regeneration of multilineage hematopoiesis from primitive cells in normal human bone marrow transplanted into immunodeficient mice.. Blood.

[5] Larochelle A, Vormoor J, Hanenberg H, Wang JC, Bhatia M (1996). Identification of primitive human hematopoietic cells capable of repopulating NOD/SCID mouse bone marrow: implications for gene therapy.. Nat Med.

[6] Kalberer CP, Siegler U, Wodnar-Filipowicz A (2003). Human NK cell development in NOD/SCID mice receiving grafts of cord blood CD34+ cells.. Blood.

[7] Ito M, Hiramatsu H, Kobayashi K, Suzue K, Kawahata M (2002). NOD/SCID/gamma(c)(null) mouse: an excellent recipient mouse model for engraftment of human cells.. Blood.

[8] Goldman JP, Blundell MP, Lopes L, Kinnon C, Di Santo JP (1998). Enhanced human cell engraftment in mice deficient in RAG2 and the common cytokine receptor gamma chain.. Br J Haematol.

[9] Legrand N, Weijer K, Spits H (2006). Experimental models to study development and function of the human immune system in vivo.. J Immunol.

[10] Traggiai E, Chicha L, Mazzucchelli L, Bronz L, Piffaretti JC (2004). Development of a human adaptive immune system in cord blood cell-transplanted mice.. Science.

[11] Gimeno R, Weijer K, Voordouw A, Uittenbogaart CH, Legrand N (2004). Monitoring the effect of gene silencing by RNA interference in human CD34+ cells injected into newborn RAG2−/− gammac−/− mice: functional inactivation of p53 in developing T cells.. Blood.

[12] Huntington ND, Legrand N, Alves NL, Jaron B, Weijer K (2009). IL-15 trans-presentation promotes human NK cell development and differentiation in vivo.. J Exp Med.

[13] Ruggeri L, Capanni M, Urbani E, Perruccio K, Shlomchik WD (2002). Effectiveness of donor natural killer cell alloreactivity in mismatched hematopoietic transplants.. Science.

[14] Miller JS, Soignier Y, Panoskaltsis-Mortari A, McNearney SA, Yun GH (2005). Successful adoptive transfer and in vivo expansion of human haploidentical NK cells in patients with cancer.. Blood.

[15] Ortaldo JR, Winkler-Pickett RT, Yagita H, Young HA (1991). Comparative studies of CD3− and CD3+ CD56+ cells: examination of morphology, functions, T cell receptor rearrangement, and pore-forming protein expression.. Cell Immunol.

[16] Gritzapis AD, Dimitroulopoulos D, Paraskevas E, Baxevanis CN, Papamichail M (2002). Large-scale expansion of CD3(+)CD56(+) lymphocytes capable of lysing autologous tumor cells with cytokine-rich supernatants.. Cancer Immunol Immunother.

[17] Imai K, Matsuyama S, Miyake S, Suga K, Nakachi K (2000). Natural cytotoxic activity of peripheral-blood lymphocytes and cancer incidence: an 11-year follow-up study of a general population.. Lancet.

[18] Yu H, Fehniger TA, Fuchshuber P, Thiel KS, Vivier E (1998). Flt3 ligand promotes the generation of a distinct CD34(+) human natural killer cell progenitor that responds to interleukin-15.. Blood.

[19] Carayol G, Robin C, Bourhis JH, Bennaceur-Griscelli A, Chouaib S (1998). NK cells differentiated from bone marrow, cord blood and peripheral blood stem cells exhibit similar phenotype and functions.. Eur J Immunol.

[20] Mrozek E, Anderson P, Caligiuri MA (1996). Role of interleukin-15 in the development of human CD56+ natural killer cells from CD34+ hematopoietic progenitor cells.. Blood.

[21] Woo SY, Jung YJ, Ryu KH, Park HY, Kie JH (2003). In vitro differentiation of natural killer T cells from human cord blood CD34+ cells.. Br J Haematol.

[22] Wallace ME, Smyth MJ (2005). The role of natural killer cells in tumor control–effectors and regulators of adaptive immunity.. Springer Semin Immunopathol.

[23] Smyth MJ, Godfrey DI, Trapani JA (2001). A fresh look at tumor immunosurveillance and immunotherapy.. Nat Immunol.

[24] Smyth MJ, Thia KY, Street SE, Cretney E, Trapani JA (2000). Differential tumor surveillance by natural killer (NK) and NKT cells.. J Exp Med.

[25] Baxevanis CN, Gritzapis AD, Papamichail M (2003). In vivo antitumor activity of NKT cells activated by the combination of IL-12 and IL-18.. J Immunol.

[26] Colucci F, Caligiuri MA, Di Santo JP (2003). What does it take to make a natural killer?. Nat Rev Immunol.

[27] Dewan MZ, Terunuma H, Toi M, Tanaka Y, Katano H (2006). Potential role of natural killer cells in controlling growth and infiltration of AIDS-associated primary effusion lymphoma cells.. Cancer Sci.

[28] Guimaraes F, Guven H, Donati D, Christensson B, Ljunggren HG (2006). Evaluation of ex vivo expanded human NK cells on antileukemia activity in SCID-beige mice.. Leukemia.

[29] Langhans B, Ahrendt M, Nattermann J, Sauerbruch T, Spengler U (2005). Comparative study of NK cell-mediated cytotoxicity using radioactive and flow cytometric cytotoxicity assays.. J Immunol Methods.

[30] Whiteside TL, Herberman RB (1995). The role of natural killer cells in immune surveillance of cancer.. Curr Opin Immunol.

[31] Macchiarini F, Manz MG, Palucka AK, Shultz LD (2005). Humanized mice: are we there yet?. J Exp Med.

[32] Smyth MJ, Cretney E, Kershaw MH, Hayakawa Y (2004). Cytokines in cancer immunity and immunotherapy.. Immunol Rev.

[33] Williams NS, Moore TA, Schatzle JD, Puzanov IJ, Sivakumar PV (1997). Generation of lytic natural killer 1.1+, Ly-49− cells from multipotential murine bone marrow progenitors in a stroma-free culture: definition of cytokine requirements and developmental intermediates.. J Exp Med.

[34] Kennedy MK, Glaccum M, Brown SN, Butz EA, Viney JL (2000). Reversible defects in natural killer and memory CD8 T cell lineages in interleukin 15-deficient mice.. J Exp Med.

[35] Lodolce JP, Boone DL, Chai S, Swain RE, Dassopoulos T (1998). IL-15 receptor maintains lymphoid homeostasis by supporting lymphocyte homing and proliferation.. Immunity.

[36] Ljunggren HG, Karre K (1990). In search of the ‘missing self’: MHC molecules and NK cell recognition.. Immunol Today.

[37] Bix M, Liao NS, Zijlstra M, Loring J, Jaenisch R (1991). Rejection of class I MHC-deficient haemopoietic cells by irradiated MHC-matched mice.. Nature.

[38] Liao NS, Bix M, Zijlstra M, Jaenisch R, Raulet D (1991). MHC class I deficiency: susceptibility to natural killer (NK) cells and impaired NK activity.. Science.

[39] Yokoyama WM, Kim S (2006). Licensing of natural killer cells by self-major histocompatibility complex class I.. Immunol Rev.

[40] Kim S, Poursine-Laurent J, Truscott SM, Lybarger L, Song YJ (2005). Licensing of natural killer cells by host major histocompatibility complex class I molecules.. Nature.

[41] Anfossi N, Andre P, Guia S, Falk CS, Roetynck S (2006). Human NK cell education by inhibitory receptors for MHC class I.. Immunity.

[42] Sun JC, Lanier LL (2008). Cutting edge: viral infection breaks NK cell tolerance to “missing self”.. J Immunol.

[43] Kamel-Reid S, Letarte M, Sirard C, Doedens M, Grunberger T (1989). A model of human acute lymphoblastic leukemia in immune-deficient SCID mice.. Science.

[44] Feuer G, Stewart SA, Baird SM, Lee F, Feuer R (1995). Potential role of natural killer cells in controlling tumorigenesis by human T-cell leukemia viruses.. J Virol.

[45] Crowe NY, Smyth MJ, Godfrey DI (2002). A critical role for natural killer T cells in immunosurveillance of methylcholanthrene-induced sarcomas.. J Exp Med.

[46] Kawano T, Cui J, Koezuka Y, Toura I, Kaneko Y (1998). Natural killer-like nonspecific tumor cell lysis mediated by specific ligand-activated Valpha14 NKT cells.. Proc Natl Acad Sci U S A.

[47] Nieda M, Nicol A, Koezuka Y, Kikuchi A, Lapteva N (2001). TRAIL expression by activated human CD4(+)V alpha 24NKT cells induces in vitro and in vivo apoptosis of human acute myeloid leukemia cells.. Blood.

[48] Taniguchi M, Seino K, Nakayama T (2003). The NKT cell system: bridging innate and acquired immunity.. Nat Immunol.

