# Supplementary figures and images for: Development of Functional Human NK Cells in an Immunodeficient Mouse Model with the Ability to Provide Protection against Tumor Challenge
Source: PLoS One. 2009 Dec 21;4(12):e8379. doi: 10.1371/journal.pone.0008379 (PMC2793015; doi:10.1371/journal.pone.0008379)

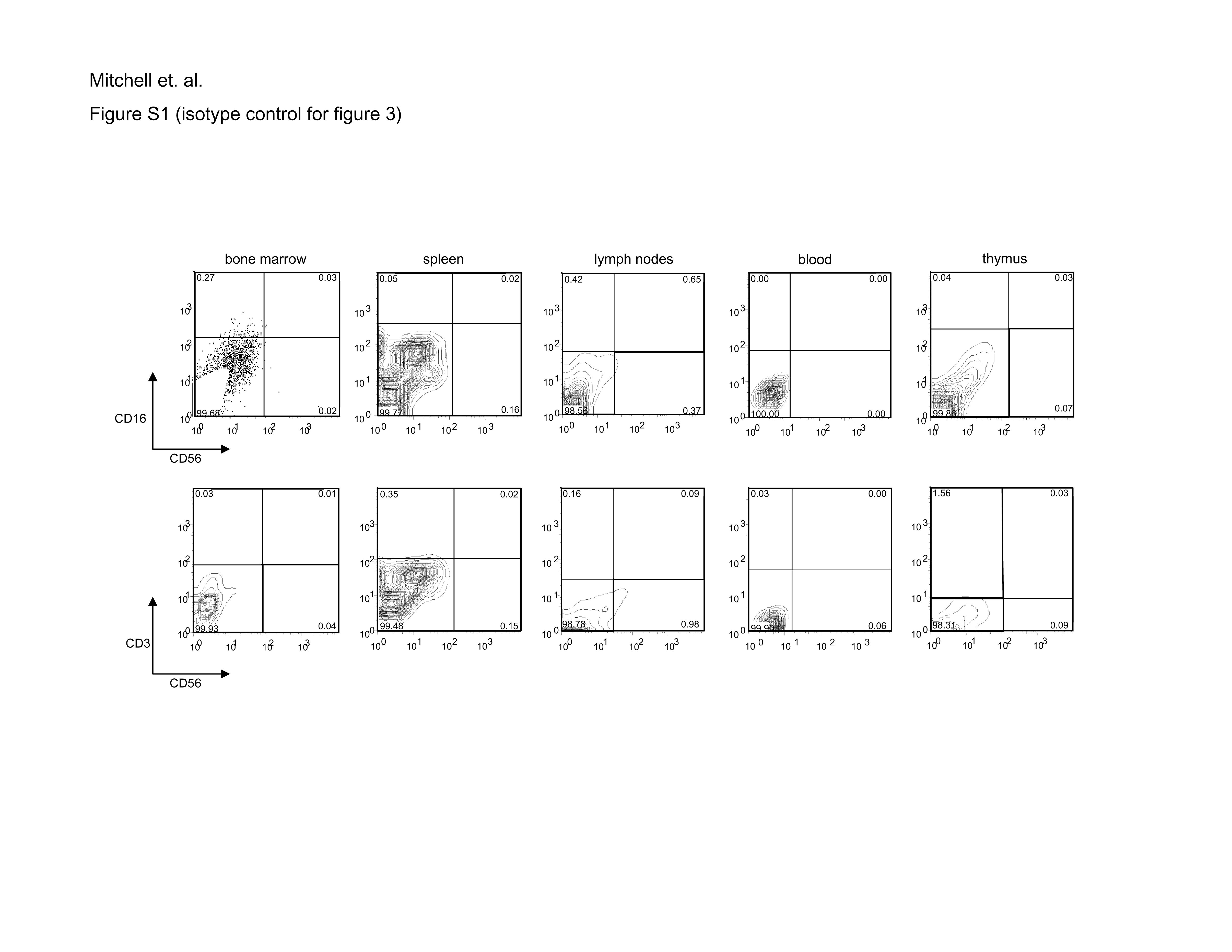

Supplement: Figure S1 — Isotype control for Figure 3 (1.16 MB TIF) [file pone.0008379.s001.tif]

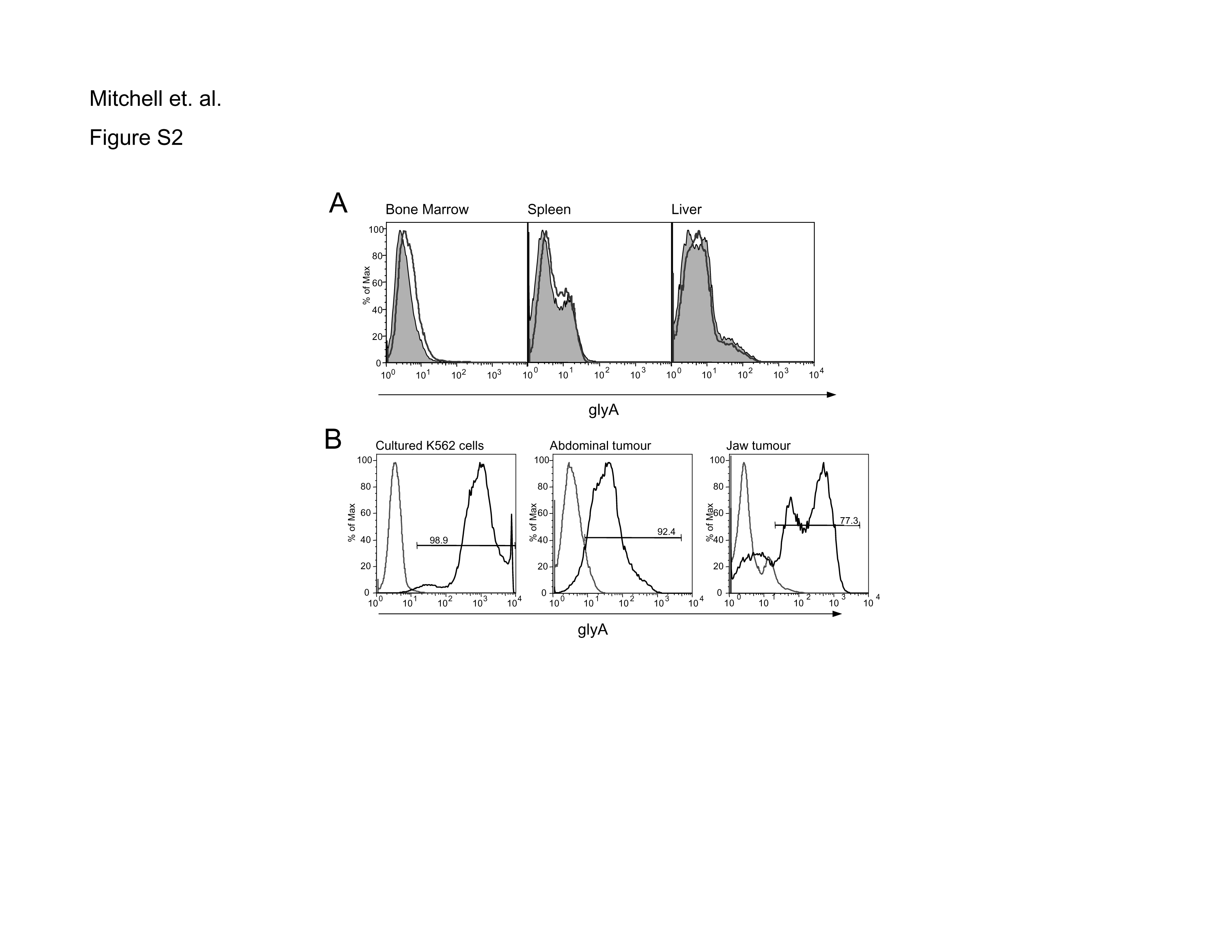

Supplement: Figure S2 — (A) Reconstituted mice that survived K562 challenge completely cleared the tumor cells when examined 90 days after challenge. Lightweight line represents BALB/c Rag2−/−/gc−/− mouse not injected with K562 cells. (B) Cultured K562 cells and cells taken from solid tumors at end point were also stained for glyA. Lightweight line represents isotype control. (0.45 MB TIF) [file pone.0008379.s002.tif]
